# Mobile Phone Technology for Preventing HIV and Related Youth Health Problems, Sexual Health, Mental Health, and Substance Use Problems in Southwest Uganda (Youth Health SMS): Protocol for a Pilot Randomized Controlled Trial

**DOI:** 10.2196/49352

**Published:** 2023-12-19

**Authors:** Philip Kreniske, Olive Imelda Namuyaba, Robert Kasumba, Phionah Namatovu, Fred Ssewamala, Gina Wingood, Ying Wei, Michele L Ybarra, Charlotte Oloya, Costella Tindyebwa, Christina Ntulo, Vincent Mujune, Larry W Chang, Claude A Mellins, John S Santelli

**Affiliations:** 1 Community Health and Social Sciences Department Graduate School of Public Health and Health Policy City University of New York New York, NY United States; 2 International Center for Child Development Masaka Uganda; 3 Washington University in St Louis St Louis, MO United States; 4 Department of Sociomedical Sciences Mailman School of Public Health Columbia University New York, NY United States; 5 Department of Biostatistics Mailman School of Public Health Columbia University New York, NY United States; 6 Center for Innovative Public Health Research San Clemente, CA United States; 7 StrongMinds Uganda Kampala Uganda; 8 Malachite Center for Mental Health Kampala Uganda; 9 Department of Epidemiology School of Medicine Johns Hopkins Bloomberg School of Public Health Baltimore, MD United States; 10 HIV Center for Clinical and Behavioral Studies New York State Psychiatric Institute and Columbia University New York, NY United States; 11 Heilbrunn Department of Population and Family Health Mailman School of Public Health Columbia University New York, NY United States

**Keywords:** adolescence, PrEP, pre-exposure prophylaxis, HIV, mental health, substance use, sexual health, mobile phones, randomized controlled trial, adaptation, Uganda

## Abstract

**Background:**

East and Southern Africa have the highest HIV incidence and prevalence in the world, with adolescents and young adults being at the greatest risk. Despite effective combination prevention tools, including the recently available pre-exposure prophylaxis (PrEP), HIV incidence among adolescents and young adults in Uganda remains high, and PrEP use remains low. Mental health and substance use (behavioral health) play a role in sexual behavior and decision-making, contributing to an increase in the risk for acquiring HIV. Interventions that target multiple HIV risk factors, including sexual and mental health and problematic substance use, are crucial to ending the HIV epidemic. Yet few interventions addressing HIV related health disparities and comorbidities among adolescents and young adults in East and Southern Africa currently exist.

**Objective:**

This study aims to evaluate the acceptability and feasibility of Kirabo, an SMS text message intervention informed by the information, motivation, and behavior model and to be disseminated through secondary schools. The study will gather preliminary estimates of Kirabo’s effectiveness in increasing HIV testing and linking users to mental health counselors.

**Methods:**

We identified Mobile 4 Reproductive Health for adaptation using the assessment, decision, administration, production, topical experts, integration, training, testing (ADAPT-ITT) framework. Mobile 4 Reproductive Health is an evidence-based automated 2-way SMS text messaging and interactive voice response platform that offers sexual and reproductive health information and links users to HIV clinics in East Africa. Through ADAPT-ITT we refined our approach and created Kirabo, an SMS text message–based intervention for linking adolescents and young adults to health services, including HIV testing and mental health counseling. We will conduct a 2-arm randomized controlled trial in Masaka, Uganda. Adolescents (N=200) will be recruited from local schools. Baseline sociodemographic characteristics, HIV test history, and behavioral health symptoms will be assessed. We will evaluate acceptability and feasibility using surveys, interviews, and mobile phone data. The preliminary efficacy of Kirabo in increasing HIV testing and linking users to mental health counselors will be evaluated immediately after the intervention and at the 3-month follow-up. We will also assess the intervention’s impact on self-efficacy in testing for HIV, adopting PrEP, and contacting a mental health counselor.

**Results:**

Intervention adaptation began in 2019. A pretest was conducted in 2021. The randomized controlled trial, including usability and feasibility assessments and effectiveness measurements, commenced in August 2023.

**Conclusions:**

Kirabo is a tool that assists in the efforts to end the HIV epidemic by targeting the health disparities and comorbidities among adolescents in Uganda. The intervention includes local HIV clinic information, PrEP information, and behavioral health screening, with referrals as needed. Increasing access to prevention strategies and mitigating factors that make adolescents and young adults susceptible to HIV acquisition can contribute to global efforts to end the HIV epidemic.

**Trial Registration:**

ClinicalTrials.gov NCT05130151; https://clinicaltrials.gov/study/NCT05130151

**International Registered Report Identifier (IRRID):**

DERR1-10.2196/49352

## Introduction

### Background

Globally, adolescents and young adults aged 10 to 24 years represent approximately 30% of people living with HIV. East and Southern Africa (ESA) have the highest HIV incidence in the world, with adolescents and young adults being at the greatest risk [1-3]. AIDS is the leading cause of death for adolescents and young adults in the region and the second leading cause of death for adolescents and young adults worldwide [4,5]. Despite combination prevention efforts, including pre-exposure prophylaxis (PrEP) [6], HIV incidence among adolescents and young adults remains high [1], and PrEP use remains low [7]. Further, in part owing to the lack of access to information and services related to mobility, the uptake of antiretroviral therapy among adolescents and young adults in Uganda living with HIV is considerably lower than that among adults in Uganda living with HIV [8-10].

Mental health and substance use affect decisions regarding sexual and reproductive health (SRH) and can increase the risk for acquiring HIV infection [11-13]. It is estimated that mental illness contributes to an estimated 4- to 10-fold increase in the risk for acquiring HIV [14-17]. In Uganda, alcohol is the primary substance used, and the country has one of the highest per capita alcohol consumption rates in ESA [18,19]. Thus, in the proposed protocol, we primarily focus on alcohol consumption. It is widely recognized that designing interventions for addressing multiple HIV risk factors, including sexual and mental health and problematic substance use, is crucial to ending the HIV epidemic, yet few such interventions currently exist for adolescents and young adults in ESA [20]. Integrating screening for mental health and alcohol use problems with appropriate referral mechanisms into existing SRH interventions may be a feasible first step toward addressing this gap.

In Uganda, household mobile phone ownership has risen dramatically over the last 15 years, from approximately 10% in 2004 to 74% in 2020, yet few people own smartphones [21,22], with a similar pattern in the specific region of study [23,24]. SMS text messages can be sent or received using any mobile phone, and SMS text messaging is the most widespread means of communication in ESA [25,26]. Systematic reviews have indicated that SMS text messaging is a feasible and acceptable method for delivering health interventions [27] focused on substance use [28], mental health [29], and HIV prevention and care [30,31]. Mobile phone–based HIV interventions in ESA are well represented in the literature. However, most mobile phone–based interventions that focus on mental health and substance use have been implemented in Europe and the United States [27-29].

### This Study

This study focuses on adolescents aged 15 to 19 years who are in secondary school in the greater Masaka region of Uganda. This is a critical developmental time for intervention, as many adolescents within this age range begin to leave school, search for work, and engage in sex and are thus at an increased risk for HIV infection [32-36]. This is also the age when, if at risk, symptoms of mental disorders typically emerge [37-40]. Consequently, this stage of adolescence is a critical time for preventive interventions, and our preliminary qualitative work shows the importance of mobile phones for finding sexual partners and the as-yet-underused potential for mobile phones to promote health [23]. As noted earlier, mental health and alcohol abuse are associated with an increased risk for HIV infection. Thus, screening adolescents with mental health or alcohol use problems and linking them to counselors may help address these factors and reduce the risk for HIV infection. We identified the local mental health partner StrongMinds (authors CO, CT, CN, and VM). StrongMinds is an international nongovernmental organization in Uganda that offers free evidence-based, telehealth-based group interpersonal therapy [41-47].

Through our formative work described in the subsequent section, we used the ADAPT-ITT (assessment, decision, administration, production, topical experts, integration, training, testing) [48] framework to adapt and update a mobile phone–based SRH intervention. Our adapted intervention, named Kirabo, is also informed by the information, motivation, and behavior (IMB) model [49] and uses automated SMS text messaging to link adolescents to HIV testing and mental health counselors. Through the proposed research, we will evaluate the feasibility and acceptability of Kirabo and generate the preliminary estimates of Kirabo’s efficacy in HIV testing. Adolescents today rely heavily on mobile phones for information and services; therefore, we believe that the proposed intervention could be applied and adapted across ESA and potentially in other underresourced settings [21,50,51].

## Methods

### Formative Work

We used the ADAPT-ITT [48] framework (Table 1) to adapt an existing evidence-based SRH mobile phone intervention, Mobile 4 Reproductive Health (m4RH), originally designed by FHI 360 [52-55]. Through our formative work, including discussions with our in-country research team (RK, OIN, and PN), we named our adapted intervention *Kirabo*, a Luganda-language gender neutral name that means “gift.” In the subsequent section, a detailed description of the adaptation process is provided, including our summary of prior findings and context, a description of the Kirabo pretest, and the proposed design of our randomized controlled trial (RCT) pilot study. Researchers at the HIV Center for Clinical and Behavioral Studies at the New York State Psychiatric Institute (NYSPI) and Columbia University (CU), in collaboration with researchers at the Ugandan office of the International Center for Child Health and Development (ICHAD) at the University of Washington in St Louis, conducted this formative work. Study participants were recruited from secondary schools in southwest Uganda, one of the regions hardest hit by HIV [1]. This region is bordered by Lake Victoria. Most of the population (80%) lives in rural communities and engages in small-scale agriculture. Most of the population (75%) is aged under 30 years [56], and the Ugandan government policy supports HIV testing and PrEP for adolescents, whom they have designated a *priority population* [6]. In the region of study, HIV clinics provide free HIV testing, PrEP, and antiretroviral treatment, although the uptake of these services among adolescents and young adults is persistently low and HIV incidence has remained high [1,6,7,57].

**Table 1:** ADAPT-ITT^a^ phases.

Phase	Methodology
1. Assessment (formative)	Analyses of quantitative and qualitative secondary data
2. Decision	Publish formative findings Submit IRB^b^ protocol
3. Administration	Administer pretest in Uganda (24 adolescents and young adults)
4. Production	Produce mobile phone–based EBI^c^ draft 1
5. Topical experts	Identify and review “product” with experts
6. Integration	Integrate expert input to create pilot-ready EBI
7. Training	Train staff to implement EBI draft 2
8. Testing and evaluation	Pilot study Initiate evaluation Mixed methods: interviews, pre-post surveys, and mobile phone data

^a^ADAPT-ITT: assessment, decision, administration, production, topical experts, integration, training, testing.

^b^IRB: institutional review board.

^c^EBI: evidence-based intervention.

### Phases 1 and 2: Assessment and Decision

#### Overview

We first conducted formative work that involved secondary data analysis of both quantitative and qualitative data to assess how mobile phones may be associated with health risks and may also present opportunities for intervention [23,58,59]. Through our longitudinal analyses among youths in southern Uganda, we found that for adolescents and young adults, mobile phone ownership was associated with social and health behaviors; adolescents and young adults who owned phones were more likely to have multiple and concurrent sexual partners, and adolescent and young adult men who owned phones were more likely to be circumcised [23,24,34,58]. Furthermore, our mixed methods analysis demonstrated how mobile phones were a key tool that adolescents and young adults used to make social and economic connections. In qualitative interviews, adolescents and young adults also expressed an unmet need for mobile phones to provide health information [23]. Adolescents and young adults in southern Uganda are highly mobile, as structural factors, such as the lack of available employment, force adolescents and young adults to move after they leave school and search for work [34]. Mobility makes it difficult to track adolescents and young adults for recommended health care services [8], and mobility is also associated with a greater risk of HIV infection [32-34]. Thus, mobile phones present an opportunity to reach adolescents and young adults with high mobility [25,26,34,50]. We identified m4RH as a candidate for adaptation. m4RH is an evidence-based [52-55] automated 2-way SMS text messaging and interactive voice response (IVR) platform that offers SRH information and links users to HIV clinics in East Africa [52-55]. IVR is an automated technology that allows incoming callers to access information via a voice response system of prerecorded messages. Communicating via the platform is free in Uganda and across East Africa owing to existing agreements between FHI 360, which designed the original m4RH platform, and the regional mobile phone service provider Airtel. However, m4RH was not specifically tailored to adolescents, and it did not include PrEP information, nor did it include any behavioral health elements.

#### The IMB Model

The IMB model [49] guided our design of new content for the adapted intervention. The IMB model posits that information (in this project, communicated through interactive SMS text messages), motivational factors, and behavioral skills (measured through self-efficacy and intentions) influence the adoption of a new behavior (HIV testing, PrEP uptake, and mental health counseling). Our adapted SMS text messages, beyond communicating basic facts, were organized through narratives, which appealed to emotions, imagination, and value systems [60-63]. Narrative components influence how people think, behave, and develop lasting health-related behaviors and, therefore, influence both information and motivational factors. In the IMB model, *motivational factors* include *positive outcome expectancies*, for example, starting PrEP will enable me to achieve future goals by protecting me from getting HIV; *negative outcome expectancies* are undesirable consequences (eg, HIV infection). Motivational factors that may be enhanced by engaging with the mobile phone platform include *perceived risk*; perceptions of *peer norms*, that is, whether a new behavior is perceived positively or negatively by important referents, such as partners, other women, or family; and *barriers to and facilitators* of adopting preventive behaviors*.* The IMB model also highlights the importance of increasing *behavioral skills* and a sense of self-efficacy in adopting prevention practices and persisting despite barriers. In short, self-efficacy is a person’s confidence in their ability and skills to perform a behavior [64]. We posit that SMS text messaging via the platform will increase adolescents’ self-efficacy by providing them with simple and direct information (including information about HIV prevention skills, clinic location and contact numbers, and information about behavioral health counselors), thus promoting their belief that they can perform the target behavior successfully (eg, going to the clinic and taking an HIV test).

#### Three Main Modifications

**Figure 1:**
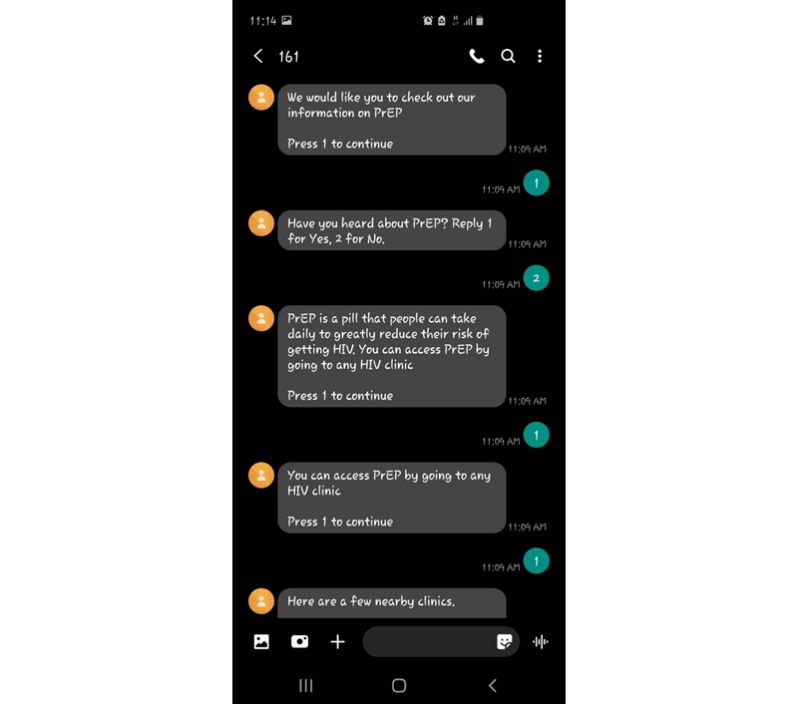
Pre-exposure prophylaxis (PrEP) messages.

In Kirabo, we made the following three main modifications to the existing m4RH approach:

1. In prior work, m4RH was disseminated via radio and magazine advertisements and at family planning clinics [52,53,55,65]. To ensure that we reach the target population of adolescents for Kirabo, we partnered with local schools and will disseminate Kirabo at the schools or via SMS text messages. Our researchers will deliver an IMB-informed oral script, describing Kirabo in person at local schools and further disseminating Kirabo via flyers.
2. When m4RH was designed, PrEP was not available in sub-Saharan Africa. Thus, in Kirabo, we added and updated the existing m4RH HIV prevention menu to include the current information on PrEP and the location of local clinics. To create PrEP messages, we used text from the Centers for Disease Control and Prevention’s (CDC) informational materials and published qualitative research studies [66-68]. Figure 1 displays an example of PrEP messages from Kirabo, with the numbers indicating user responses and menu selections. In Figure 1, Kirabo asks the adolescent the following question: “have you heard of PrEP? Reply 1 for Yes, 2 for No.” The adolescent then replies “2,” indicating that they had not previously heard of PrEP and thus triggering Kirabo to begin to describe PrEP. Furthermore, we added additional details, such as local landmarks, directions, and contact numbers for local HIV clinics. As part of this formative work, we contacted 6 HIV clinics in the southern Ugandan region. Of the 6 clinics, 5 confirmed that they were currently able to prescribe PrEP to adolescents according to the national guidelines and had ample PrEP medication in supply [69]. The clinic location and contact information were included in the adapted intervention.
3. Finally, because we know that mental health is critical to HIV prevention efforts, in Kirabo, we additionally focus on behavioral health. Adolescents will complete screeners, which are then used to tailor the SMS text messages they receive.

For mental health, we will measure depression using the 4-item Patient Health Questionnaire (PHQ-4), which includes the first 2 questions of the 9-item Patient Health Questionnaire (PHQ-9) [70] and the first 2 questions of the 7-item Generalized Anxiety Disorder Questionnaire (GAD)-7 for assessing anxiety [71]. For example, question 1 of the PHQ reads, “over the last two weeks, how often have you had little interest or pleasure in doing things?” Possible responses range from “not at all (0)” to “nearly every day (3).” Individuals who score ≥1 on the first 2 questions of the PHQ-9 or GAD-7 will be directed to complete the full measure. If the respondents score 0 on the first 2 questions of these measures, the system will respond with a brief message thanking them for their time. To be cautious and consistent with StrongMinds’ approach, we will use a cutoff point of 8 on the PHQ-9 to determine a referral to a StrongMinds counselor. Additional information regarding these measures is provided in Table 2.

**Table 2:** Additional information regarding measures and their times of collection.

Construct		Measures	Time
**Behavioral, mental health, and alcohol use measures**			
	Demographics	Items developed for previous Suubi studies [72-74], including age, sex, parents’ occupation, household size, orphanhood status, primary caregiver, and community of origin	Baseline
	COVID-19 experiences	CASPE^a^ (adapted from Suubi+Adherence [74] and the study by Ladouceur [75])	Baseline
	HIV testing	Self-report of HIV test (yes/no; past year [at baseline], during the study period [immediately after the intervention], and past 3 months [at follow-up]) confirmed with proof of results from the clinic using measures and procedures adapted from Suubi studies [72-74]	Baseline, immediately after the intervention, and 3-month follow-up
	Intention to test for HIV	Questions adapted from a study of secondary school students in Uganda [76]	Baseline, immediately after the intervention, and 3-month follow-up
	Self-efficacy	Questions adapted from a study with secondary school students in Uganda [76] and a study with high school students in the United States [77], including questions regarding self-efficacy in testing for HIV, trying PrEP^b^, using condoms consistently, contacting a behavioral health counselor	Baseline, immediately after the intervention, and 3-month follow-up
	Current HIV prevention strategies	This measure includes items developed for previous Suubi studies [72,73] in Uganda (in addition to a question regarding PrEP; range 5-25; 5 question items; α=.70), for example, “I think it is very important to use condoms every time one has sex,” “as a teenager I think AIDS is a threat to my health,” and “I think all people my age who have sex should use condoms.” Scores range from 4: strongly agree; 3: somewhat agree; 2: somewhat disagree; to 1: strongly disagree.	Baseline, immediately after the intervention, and 3-month follow-up
	HIV-related knowledge	This measure includes items from previous Suubi studies [72,73] in Uganda; this measure includes 13 items about HIV infection and prevention mechanisms (range 0-16; 16 question items; α=.83), for example, “HIV can be passed by an infected person even though that person is not feeling sick.” Possible scores are 1: true; 2: false; and 3: don't know or 1: not sure; 2: unsafe; and 3: safe.	Baseline, immediately after the intervention, and 3-month follow-up
	Sexual risk behaviors	This measure includes items from previous Suubi studies [72,73] in Uganda (range: 5-25; 5 question items; α=.76), for example, “I believe it’s OK to have sex without protection with someone you know.” Scores range from 1: never; 2: sometimes; 3: about half of the time; 4: most of the time; to 5: always.	Baseline, immediately after the intervention, and 3-month follow-up
	PrEP knowledge	Adapted 13-item true-false questionnaire on PrEP knowledge [78,79] including questions such as “PrEP is a pill you can take to prevent HIV” and “PrEP is only for people who are HIV negative”	Baseline, immediately after the intervention, and 3-month follow-up
	Depressive symptomatology	PHQ-9^c^ [70], a standardized 9-item self-report instrument used to assess depression, is used. Participants rate the frequency of their symptoms over the past 2 weeks. Items are rated on a 4-point scale, ranging from 0 (not at all) to 3 (nearly every day), and scale scores range from 0 to 27. PHQ-9 scores of 5 to 9 represent minimal to mild depression and 10 to 20 represent moderate to severe depression. A score ≥10 has a sensitivity of 88% and a specificity of 88% for major depression.	Baseline and immediately after the intervention
	Anxiety	GAD-7^d^ [71], is a standardized instrument that asks participants to rate their symptoms over the past 2 weeks on a 4-point scale ranging from 0 (not at all) to 3 (nearly every day), is used. Overall scores for the GAD-7 range from 0 to 21. At a cutoff point of 10 (indicative of clinically significant symptoms), the sensitivity and specificity of the scale equal 89% and 82%, respectively. The GAD-7 has a Cronbach α of.92, indicating good internal consistency, and an intraclass correlation of 0.83, indicating good procedural validity.	Baseline and immediately after the intervention
	Alcohol use disorder screen	AUDIT-C^e^ [80,81], a 3-item screener excerpted from the full standardized 10-item AUDIT tool for assessing hazardous alcohol consumption, is used. Items are scored on a 5-point scale for a possible score of 0 to 12. The higher the score, the higher the risk of hazardous alcohol consumption. AUDIT-C scores of ≥4 in men and ≥3 in women are considered positive for hazardous alcohol consumption.	Baseline and immediately after the intervention
	Reported (and confirmed) sessions with mental health counselor	Self-report and local school and clinic records	Baseline and immediately after the intervention
	Peer messaging	Self-report of messages shared with peers	Baseline and immediately after the intervention
**Technological, feasibility, and acceptability measures**			
	Survey of acceptability	This measure, adapted from CyberSenga [82] (a mobile health HIV study with youths in Uganda), is a 12-item survey with a Likert scale of 1 to 3 (disagree, somewhat agree, and strongly agree).	Immediately after the intervention
	Number of messages viewed	Retrieved via user database consistent with previous m4RH evaluations [54]	Continuous
	Type of messages (regarding sexual health, mental health, and alcohol use)	Retrieved via user database consistent with previous m4RH evaluations [54]	Continuous
	Mental health and alcohol use screens completed (AUDIT, GAD-7, and PHQ-9)	Retrieved via user database	Continuous
	HIV clinic locator (number of times used)	Retrieved via user database	Continuous
	Feasibility	Enrollment of recruited adolescents	Immediately after the intervention

^a^CASPE: COVID-19 Adolescent Symptom & Psychological Experience Questionnaire.

^b^PrEP: pre-exposure prophylaxis.

^c^PHQ-9: 9-item Patient Health Questionnaire.

^d^AUDIT-C: Alcohol Use Disorders Identification Test–Consumption.

^e^GAD-7: 7-item Generalized Anxiety Disorder Questionnaire.

Similarly, the GAD-7 [71] will be used to screen and measure the severity of generalized anxiety symptoms among participants. Through Kirabo, we will administer the first 2 questions of the GAD-7. Table 2 provides additional information regarding this measure. To be cautious and consistent with StrongMinds’ approach, we will use a cutoff point of 8 to determine a referral to a StrongMinds counselor.

The 3-item Alcohol Use Disorders Identification Test–Consumption (AUDIT-C) [80] will be used to measure hazardous alcohol use (Table 2). Similar brief measures have been used and validated globally, specifically in East Africa and Uganda [18,83-89]. To improve user experience and consistent with the prior work described subsequently, screener length will be determined by an adolescent’s response to initial questions. Table 2 provides additional details regarding the AUDIT-C [90].

In our initial version of Kirabo, if an adolescent screened positive for symptoms of depression, anxiety, or hazardous alcohol use, the adolescent was provided with the contact number of our mental health partner StrongMinds.

### Phases 3 and 4: Administration of Pretest and Production

The ICHAD has research relationships with 70 secondary schools in the region, and a well-established protocol for recruitment is described subsequently. The field research team (RK, OIN, and PN) worked primarily out of the ICHAD offices in Masaka, Uganda. ICHAD researchers distributed flyers to students and staff, noting that adolescents aged 15 to 19 years may be eligible to participate. Research team members with no relationship with students conducted the recruitment. Further, students did not receive any class credit or extra credit for participating. Students were compensated for their time at rates consistent with the ICHAD policies and in accordance with Ugandan and US ethics board guidance. Adolescents were compensated 8500 Uganda shillings (US $2.25) for participating in the mobile phone–based portion of the research, and adolescents who joined the focus groups were offered an additional 20,000 Uganda shillings (US $5.30). Teachers were not informed about which students were contacted and consented/assented to participate. We will use similar procedures to recruit for the RCT described subsequently. That said, if the current COVID-19 protocols or other factors result in the school not currently being in session, a head teacher may provide researchers with a list of students who have expressed interest and meet the age eligibility for a final screen by the research team.

To conduct the pretest, we recruited 24 adolescents (aged 15 to 19 years) from 2 secondary schools in southern Uganda. Over a 1-week period, the adolescents were prompted via a daily automated SMS text message, amounting to 5 prompts per week, to interact with Kirabo. We tested the following three different methods of contacting Kirabo: (1) through IVR by dialing 161, (2) through unstructured supplementary service data (USSD) by dialing *161#, or (3) through SMS text messaging by texting “Hi” to 161. IVR allows callers to access prerecorded messages through speech recognition. USSD is similar to SMS text messaging, except that the messages disappear after a texting session. Only adolescents with phone numbers registered in our study could access the messages.

Our daily automated SMS text messages described how the system could be accessed via IVR by dialing 161 or via SMS text messaging by sending “Hi” to 161. On each day of the 5-day pretest, our automated messages encouraged the adolescents to interact with a different component of the platform. Day 1 focused on HIV testing, day 2 focused on PrEP, day 3 focused on mental health, day 4 focused on alcohol use, and day 5 focused on HIV testing again. To select an option, the user should reply with a text using a keyword or code from the provided menu, for example, “press 2 for PrEP information.” The platform then automatically responds with a text or an IVR message. Adolescents could choose to interact with Kirabo in English or Luganda, the most commonly spoken local language in the region. Adolescents were advised to use their phones outside school hours, and no adolescents reported conflicts with parents or school staff regarding their use of phones in our pretest. Figure 1 shows an example of how an adolescent could move through the PrEP messages.

During the 5-day pretest, of the 24 adolescents, 22 (92%) accessed Kirabo at least once. In total, adolescents accessed Kirabo 339 times: 145 (42.8%) times through USSD, 83 (24.5%) times through SMS text messaging, and 111 (32.7%) times through IVR. SMS text messaging had the highest rate of message category completion (meaning that an adolescent viewed and responded to all messages; see Figure 1 for a PrEP example), with 83% (69/83) of the access attempts successfully running from beginning to end, compared with 58% (82/145) for USSD and 61% (68/111) for IVR.

Of the 24 adolescents, 22 (92%) completed the behavioral health screeners. The PHQ-9 scores ranged from 0 to 17 (mean 3.99, SD 3.85), and 5 (21%) adolescents scored ≥10 and thus screened positive for clinically significant symptoms of depression. Among the 24 adolescents, 1 (4%) screened positive for suicidal ideation. Our project coordinator followed up with this adolescent and, as dictated by our emergency protocol, used the Columbia Suicide Severity Rating Scale (C-SSRS) [91] to assess suicide risk. The adolescent described that they no longer experienced suicidal ideation. The project director determined that the participant was at moderate risk and then linked the adolescent to an ICHAD mental health counselor for further evaluation and support.

The GAD-7 scores ranged from 0 to 15 (mean 4.39, SD 3.77), and of the 24 participants, 5 (21%) scored ≥10 and thus screened positive for clinically significant symptoms of anxiety. In total, 3 (12%) adolescents screened positive for both symptoms of anxiety and depression. No adolescents screened positive on the AUDIT scale, with scores ranging from 0 to 4 (mean 0.5, SD 1.01).

In focus groups with 19 (79%) of the 24 adolescents, 10 (53%) preferred SMS text messaging and 9 (47%) preferred USSD, noting that SMS text messages arrived more quickly and could be revisited because they were saved on the phone. A female student aged 15 years said, “if the messages are saved, it can help me to seek clarification of what I haven’t understood. I can review those messages for me to understand them well.” None of the adolescents preferred IVR, which had the most reported issues. The adolescents explained that IVR calls frequently dropped mid-call owing to poor phone service. This would require an adolescent to call back and start at the beginning of the menus. Not surprisingly, the adolescents expressed frustration when describing this process. In addition, although using IVR was free, some adolescents assumed that calling the IVR system would cost them money. Furthermore, adolescents were hesitant to call StrongMinds counselors because they did not want to incur charges. Some adolescents noted that receiving messages after chores in the morning was preferable, whereas the majority preferred to receive messages after school and in the evening.

With regard to HIV prevention, most adolescents reflected that Kirabo helped them learn about PrEP and the health centers where they could access HIV services. As one of the adolescents succinctly explained, “it [Kirabo] is good because most of us have not been knowing what PrEP is.” Another adolescent added, “the messages I liked most were those regarding PrEP. I got more clarification about PrEP because I only knew about a tablet that you take to prevent yourself from getting infected with HIV, but I didn’t know its name.” A third adolescent described how Kirabo “directed us to the nearest health facilities where a person can easily access HIV testing and PrEP services.” These excerpts and the broader focus group discussions align with prior work showing an unmet need for mobile health HIV prevention interventions for adolescents in the region [23].

### Phases 5 to 6: Consultation With Topical Experts and Integration

In addition to weekly meetings where author 1 discussed and designed the intervention and modifications with coauthors 2, 3, and 4, the first iteration of Kirabo was presented to a group of topical experts as part of an HIV Implementation Science and Inter-Centers for AIDS Research (CFAR) Fellowship program (2022-2023; P30AI094189). In the culminating session of this 9-month training program, author 1 presented pretest findings to 30 trainees (graduate students, postdoctoral fellows, and early career faculty) and 5 faculty mentors in person (Drs Sheree Schwartz, Stefan Beral, Patrick Sullivan, and JD Smith). This presentation was given in Baltimore, Maryland, on April 5, 2022. One of the audience members suggested that given the potentially sensitive nature of the messages, we should send a preliminary greeting message with no sexual or mental health content. Another point of discussion was to consider using a shorter follow-up period of 3months as opposed to the initially planned 6 months. Comments on the presentation in combination with our pretest findings led us to make the following three key changes to Kirabo in the proposed RCT:

1. Most adolescents preferred SMS text messaging. Thus, to streamline our delivery, recruitment, and evaluation, we decided to focus on SMS text messaging in this pilot RCT. In addition, to protect privacy, we added an initial message to be sent on each day of the intervention period that asked, “is this a good time to chat?” By contrast, the initial prompt from the pilot test immediately introduced the topic of the day (HIV testing, mood, etc). If adolescents respond with “yes,” then they would be sent the follow-up message that introduces the topic of the week. If adolescents respond with “no,” they would be sent a message that says, “ok thanks, write back with yes when you are free and available.”
2. In the pretest, adolescents who screened positive for symptoms of depression, anxiety, or hazardous alcohol were provided with the contact number of the general StrongMinds counselor. As described earlier, adolescents expressed concern that they would incur charges by calling StrongMinds counselors. Therefore, we revised our methods (including language in our consent forms), and in the RCT, we will provide a daily update to StrongMinds counselors with a list of contact numbers of adolescents who screened positive for symptoms of depression, anxiety, or hazardous alcohol use.
3. On the basis of our initial focus group discussions and our presentation at the HIV Implementation Science Fellowship, we expect that a 3-month follow-up will be sufficient to determine feasibility and accessibility. In addition, a shorter follow-up period will limit the possibility of unforeseen historical or COVID-19–related interruptions.

### Phases 7 to 8: Training and RCT Pilot Study

#### Procedures

We plan to conduct a 2-arm, randomized controlled study via mobile phones among adolescents in the greater Masaka region of Uganda. We will compare Kirabo to an inactive control and evaluate whether adolescents who interacted with Kirabo had higher rates of self-reported HIV testing (yes/no; verified with the HIV clinic when possible) immediately after the intervention and at the 3-month follow-up assessment. Secondary outcomes include differences in condom use, PrEP knowledge, PrEP uptake for eligible patients, HIV-related knowledge and sexual risk behaviors, and antiretroviral treatment uptake for eligible patients. Exploratory aims include the number of participants who screen positive for symptoms of depression, anxiety, and hazardous alcohol use and the proportion of confirmed linkages to mental health counseling.

**Figure 2:**
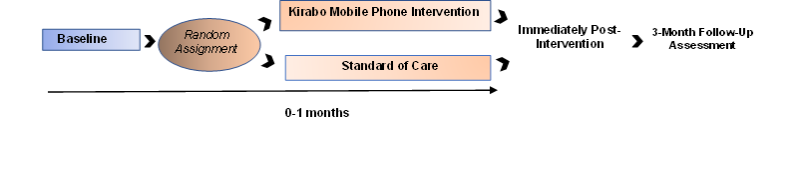
Timeline of participant involvement and measures.

Adolescents (aged 15-19 years) in the greater Masaka region in southern Uganda who own a mobile phone will be eligible for the trial. Adolescents will be involved in the trial for approximately 5 weeks with a 3-month follow-up assessment (Figure 2). After informed assent and parental permission, the baseline assessment, and confirmation of eligibility, adolescents will be randomly assigned to the Kirabo intervention group or standard of care group. Randomization will be performed in a 1:1 ratio and stratified by gender and school (to minimize contamination) with a block size of 10. We anticipate recruiting students from 20 schools. The treatment assignments will be generated using a pseudorandom number generator. An electronic list of IDs, access codes, and credentials will be provided securely to the sites.

#### Accessing Kirabo

Twice per week over the 5-week study period, Kirabo will send enrolled adolescents an SMS text message encouraging them to interact with our system. As noted earlier in the pretest, adolescents in the RCT can access Kirabo by sending the SMS text message “Hi” to #161 to initiate an interaction with Kirabo in English or Luganda. In the pretest, adolescents did not report any conflict with parents or school staff regarding their use of a mobile phone to interact with Kirabo.

### Measures

#### Overview

To evaluate the intervention’s impact, there will be 3 main assessment time points: baseline, immediately after the intervention, and 3-month follow-up. Table 2 provides a full list of measures. The primary outcome will be self-reported HIV testing, confirmed by HIV clinic records when possible. We will also measure the uptake of HIV prevention strategies (PrEP uptake and condom use) and, although we expect this to be a rare occurrence, the initiation of antiretroviral treatment. For adolescents who screen positive for behavioral health problems, we will confirm self-reports of linking to mental health care with a StrongMinds counselor.

Many of the measures have already been used, pretested, and made culturally appropriate to the Ugandan context in past studies [92,93], including current HIV prevention strategies and HIV-related knowledge. New measures, such as PrEP knowledge, will be adjusted according to the established ICHAD procedures. Specifically, to assess the uptake of HIV prevention strategies, we will compare presurvey and postsurvey scores from the current HIV prevention strategy measures that have been previously used in this region of Uganda with adolescents (Table 2) [72,73]. For questions measuring sensitive behaviors (ie, sexual risks), we will use audio computer-assisted self-interviews so that the participants may take the survey themselves on a minilaptop.

#### Behavioral Health Screeners via Mobile Phone

As in the pretest, behavioral health screeners will be delivered via adolescents’ mobile phones and automatically scored by Kirabo. In addition, we will use the same measures of behavioral health in the baseline and 3-month follow-up battery.

#### Acceptability and Feasibility Measures

All adolescents in the intervention will complete the 15-item acceptability survey adapted from prior mental, sexual, and reproductive health work among adolescents in Uganda [94]. To further evaluate feasibility, we will use paradata and text count measures previously used by FHI 360 (Table 2) [54].

In addition to the quantitative measures included in Table 2, immediately after the intervention, we will conduct 30 key informant interviews (KIIs) with participants in the intervention group. The KII guide draws from the Consolidated Framework for Implementation Research (CFIR) [95,96], with a focus on the intervention characteristics and the outer setting, and will be adapted from our pretest KII guide. For example, through our pretest and subsequent focus groups, we identified the necessary changes to our intervention based on adolescents’ descriptions of outer-setting barriers. Specifically, adolescents reported that they often experienced intermittent mobile phone service. Our earlier version of the mobile phone intervention that included IVR required callers to restart at the initial message each time they called in. Adolescents in our focus groups expressed that restarting the IVR each time a call dropped was cumbersome and frustrating. By contrast, adolescents found that SMS text messaging could be resumed wherever they had left off when their service resumed. Adolescents overwhelmingly selected SMS text messaging as their preferred means of interacting with Kirabo. Acceptability questions will probe adolescents’ comfort with content and delivery methods. All adolescents in the intervention will complete the 15-item acceptability survey adapted from prior mental, sexual, and reproductive health work among adolescents in Uganda [94]. To further evaluate feasibility, we will use paradata and text count measures previously used by FHI 360 (Table 2) [54].

### Statistical Analysis

#### Primary and Secondary Outcomes and Analysis

Summary statistics will be used to describe all metrics and compare them between the students receiving Kirabo and those in usual care at baseline, 1-month postintervention assessment, and the final 3-month follow-up assessment. Means and SDs will be used for continuous outcomes, followed by box plots, and frequency tables will be used for binary and categorical metrics. The primary analysis of this RCT will test whether postintervention HIV testing rates among the adolescents who interacted with Kirabo are significantly higher than those who received standard of care. We will use a generalized linear mixed model to determine statistical significance. In the model, the outcome is the binary indicator of whether an adolescent received an HIV test during or immediately after the intervention. We will also examine the differences between the model at the 3-month follow-up. The main predictor is the intervention assignment (Kirabo or usual care). The school will be included in the model as the random effect to adjust for within-school correlations. We will apply similar analyses to investigate the impact of Kirabo on the secondary outcomes.

Depending on the nature of the outcome, we will use different link functions in the generalized linear mixed model. We will use the identity link for continuous outcomes, the logistic link for binary outcomes, and the log function for ordinal outcomes. For incidence/frequency outcomes, a log function with adjustment for exposure time will be used. We will formally test whether the students in the 2 intervention groups have comparable baseline characteristics, including demographic factors, moderators, and variables measuring potential mechanisms of action (Table 2). If any of these variables are significantly different at baseline, they will be included in the model to adjust for their potential confounding effects.

#### Acceptability Power Analyses

The primary purpose of this pilot RCT is to determine whether Kirabo is acceptable (>70% of adolescents “agree” or “completely agree” with 70% of acceptability measure items) and feasible (enrollment >75%) in this setting. The 100 participants in the Kirabo arm will provide 80% power to detect the proportion of participants who meet the acceptability agreement threshold as significantly greater than 70% if the observed proportion of participants who meet this threshold is at least 83.5%.

#### Feasibility

The 100 participants in the Kirabo arm will provide 80% power to detect enrollment greater than 75% if the observed enrollment is at least 87.3%. These calculations assume a 1-sided significance level of 5%. In addition, we will calculate summary statistics of the technological variables to describe feasibility, including paradata (eg, number of adolescents who ever contacted Kirabo out of the total sample and messages and types of messages viewed), and to assess behavioral health, we will divide the number of counselor calls attempted by the number of counselor calls completed. These measures will objectively show whether adolescents were able to use Kirabo.

#### Effectiveness Power Analysis

There is limited information on the impact of SMS text message–based interventions on increasing HIV testing among adolescents in rural Uganda. We expect that the baseline 12-month HIV testing rate among adolescents aged 15 to 19 years will be 26% [97]. With 200 students (intervention: n=100, 50%; standard of care: n=100, 50%) from 10 schools, we have 80% power to detect an average postintervention HIV test rate of ≥46% at the significance level of.05.

#### Handling of Missing Data

If more than 10% of the observations are missing, we will use multiple imputation strategy to accommodate missing data, followed by sensitivity analysis. The inclusion of cases with partial data is an important step because the intention-to-treat approach is planned to be the main analytic strategy for the primary aim of generating preliminary estimates of Kirabo’s efficacy. If the statistical or clinical significance of treatment effects appears to differ depending on how missing data are addressed in the analyses, such findings will be reported.

#### Primary End Point Analysis for the Pilot RCT

We will recruit 200 students who own mobile phones and randomize them in a 1:1 ratio into intervention and control groups. The variables of focus are listed in Table 2. The primary end point is the binary indicator of whether there is an increase in HIV testing following the intervention. Chi-square tests will be used to determine whether intervention participants were more likely to take an HIV test in the past month. We will then build a logistic model to estimate intervention efficiency while adjusting for baseline characteristics, including baseline HIV prevention strategies, HIV knowledge, sexual risk behaviors, depression, anxiety, alcohol use, and demographics. The same analysis will be applied to the 6-month postintervention follow-up to determine the long-term effects of the intervention. These estimated parameters allow reliable estimates of sample size, power, and detectable effects for the subsequent study.

#### Secondary End Point Analysis

We will evaluate the change in behavioral health status and PrEP knowledge before and after the intervention and construct 95% CIs. Generalized linear mixed effect models will be used to reassess the intervention effect on these measurements while adjusting for the baseline characteristics age, gender, and other demographics. Finally, the same analysis will be applied to the 3-month postintervention follow-up measures to determine the long-term effects of the intervention. These estimated parameters allow reliable estimates of sample size, power, and detectable effects for the subsequent study.

#### Mixed Methods Implementation Analysis

We will use mixed methods, including systematic coding informed by CFIR [95,96], surveys, and paradata, to identify the specific elements of Kirabo that we hypothesize will be predictive of successful implementation [98]. This approach will inform the evaluation of the complex processes that motivate adolescents in underresourced settings to seek care. We will transform qualitative data into quantitative data for adolescent responses pertaining to the variables that IMB and CFIR dictate will be predictive of implementation outcomes. For instance, we will focus on constructs in adolescent KII responses that relate to perceived risks and benefits of care for mental health and alcohol use problems. In addition, for specific constructs, we will incorporate technological data, qualitative data, and referral to care data. We will then link users’ actual paradata from their texts (eg, the number of intervention messages viewed) with our adapted m4RH program to their KII responses, survey responses on the perceptions of risk behavior before and after the intervention, and service-seeking rates. This approach will offer a deeper and complex understanding of the ways in which adolescents’ beliefs impact their decision to seek care.

### Ethical Considerations

The research staff obtained written informed consent from adolescents aged ≥18 years and personal assent and permission from adult parents/guardians for adolescents aged <18 years before study enrollment. The consent processes for adults and children were performed separately to avoid coercion. Both consent and assent forms were translated into Luganda (the most widely spoken local language in the study region) and back-translated into English to ensure consistency. Both the assent and consent processes were conducted verbally in Luganda, given that some caregivers and adolescents were illiterate. The study team received training on Good Clinical Practice (GCP) so that sensitive research activities could be handled appropriately. In addition, all interviewers completed the Collaborative Institutional Training Initiative (CITI) certificate and National Institutes of Health (NIH) certificate for the protection of research participants. All study procedures were approved by the NYSPI Institutional Review Board (IRB), the home institution of the principal investigator (PI), and the in-country local ethics board of the Uganda Virus Research Institute (UVRI; reference number GC/127/835). The ethics protocol was also reviewed and approved by the Uganda National Council for Science and Technology (UNCST; reference number SS969ES). This study was registered at ClinicalTrials.gov (NCT05130151).

### Adverse Events and Safety Monitoring

The study will maintain records of adverse events and any referrals for counseling as well as copies of the consent and assent forms. All records will be maintained in a locked filing cabinet at the ICHAD Uganda field office and will be accessible only to the research team. The PI will be responsible for data security and record keeping. The data sets used for analysis will not contain any identifying information, specifically the names and addresses of the participants.

The HIV Center for Clinical and Behavioral Studies has a Performance and Safety Monitoring Board (PSMB) that consists of an independent group of experts. Currently, the members are Stephen Morin (chair), Joseph Hogan, Patricia Marshall, and Timothy Wilkin. The members of the PSMB serve in an individual capacity and provide their expertise and recommendations for studies involving human participants. The primary responsibilities of the PSMB are to (1) periodically review and evaluate the accumulated study data for participant safety; study conduct and progress; and, when appropriate, efficacy and (2) make recommendations concerning the continuation, modification, or termination of studies. The PSMB considers study-specific data as well as relevant background knowledge about the disease, test agent, or patient population under study. The PSMB has defined deliberative processes, including event triggers that would call for an unscheduled review, stopping procedures that are consistent with the protocol, unmasking (unblinding), and voting procedures. The event triggers include serious adverse events. The PSMB is also responsible for maintaining the confidentiality of its internal discussions and activities as well as the contents of reports provided to it. On November 9, 2022, Kirabo was presented to the PSMB. At that time, the PSMB had no concerns regarding the performance, monitoring, or safety of the study and recommended the continuation of the study as planned.

All study personnel based in Uganda will be trained in identifying indicators of conditions that may jeopardize the welfare of participants and the limits of confidentiality. This training, conducted by author FS and research staff, focuses on possible scenarios and key questions used to assess risk. Interview staff are trained to err on the side of caution and told to contact the project coordinator and the Masaka diocese in charge of schools, both of whom are always available, via telephone, in the event of the need to break confidentiality owing to an instance of mandatory reporting. Under the guidance of author FS, author OIN, and the parish priest, research staff are trained either to contact the police to ensure the safety of participants or, if appropriate, to have emergency personnel take the adolescent to the nearest hospital.

Adverse events will be reported according to a project protocol. In addition to the PSMB, described earlier, for this study, the PI (PK), the author FS, the project coordinator (author OIN, based in Uganda), and the parish priest (also based in Uganda) will oversee the safety and monitoring of adverse events. This group is expected to meet in person 3 to 4 times per year and will have weekly conference calls. In case of an adverse event, staff will inform the in-country project coordinator and the parish priest immediately and then the US-based PI within 24 hours (with substantial efforts made to also inform the PI immediately) of the occurrence of a possible unanticipated adverse event. Any occurrence of a possible unanticipated adverse event will be immediately reported and brought to the attention of the NYSPI IRB along with the UVRI Research Ethics Board and Ugandan National Council of Science and Technology IRB. The IRBs and PSMB will determine whether it is appropriate to stop the study protocol temporarily or will provide suggestions and modifications for the study procedures. Possible modifications may include adding new risks to the consent form and reconsenting all study participants.

Kirabo aims at HIV prevention and screening for mental health and alcohol use problems. Preliminary outcome data will be examined monthly by the PI (PK) and authors OIN, FS, JSS, and CAM. If preliminary outcome data indicate a harmful impact of the study on the participants (the adolescents), the NYSPI IRB, as well as the Uganda National Council of Science and Technology, will be notified, and it is possible that the study will be discontinued immediately. However, we do not anticipate any negative effects of participation at this time, as much of the proposed intervention is publicly available through m4RH without adverse events. In addition, findings from earlier studies guiding this application suggest an association with positive outcomes, such as increased knowledge of family planning approaches.

### Data Management, Study Oversight, and Monitoring

Human participant protection and data safety are of utmost priority. The primary risk in this study is loss of confidentiality, and there is a possible risk of psychological discomfort or distress arising from the personal and potentially sensitive nature of the information elicited during interviews or intervention sessions. The level and probability of experiencing such risks are minimal, and the monitoring plan described in this paper is commensurate with these risks. All procedures and measures were approved by the UVRI Research Ethics Board and the UNCST as well as the IRBs at the NYSPI and CU and the HIV Center for Clinical and Behavioral Studies PSMB. In addition, the Kirabo intervention was also presented to and approved by the Ugandan Ministry of Health.

## Results

Study recruitment commenced in August 2023 (N=200). Data collection and analysis are expected to be concluded by 2025, respectively. At the time of manuscript submission, no data had been analyzed. This trial was registered with ClinicalTrials.gov (NCT05130151), where the results are expected to be published from 2024 to 2025.

## Discussion

### Principal Findings

Kirabo begins to address the National Institute of Health Office of AIDS Research high priority area of reducing HIV incidence by targeting health disparities and comorbidities, as they challenge the efforts to end the epidemic. The first step in this direction is to promote HIV testing and increase mental health and alcohol use problem screening and linkage to counselors for adolescents and young adults who are symptomatic in regions with a high HIV seroprevalence and limited resources. As described in this protocol, we used the ADAPT-ITT [48] framework to adapt an existing evidence-based mobile phone–delivered sexual health program [54,65,99]. Through this process, including a pretest, we created Kirabo, a pathbreaking approach to link adolescents and young adults to local HIV clinics, PrEP, and mental health and alcohol use problem screening, with referral as needed.

Our pretest results were promising and suggest that it is possible to use school-based recruitment, and the IMB model [49] informed mobile phone SMS text messaging to integrate HIV prevention strategies and mental health care for adolescents and young adults in rural Uganda. The larger population included in our upcoming pilot RCT will provide a clearer understanding of the broad challenges that may impact feasibility and acceptability as well as initial estimates of the intervention effect size.

Mobile phones present an opportunity for contacting, providing information, and motivating adolescents and young adults to seek HIV and mental health care. Although not a panacea, mobile phones have been used for HIV-related sexual health and substance use interventions [100-103] and hold promise for adolescents and young adults who have limited access to the few and frequently overburdened mental health professionals in the region [104,105], specifically in Uganda [18,106,107]. A systematic review of digital innovations for HIV care and prevention noted the need for increased research tailored to context and population to reduce risky behaviors [30]. Furthermore, a review of mobile phone–based interventions in low- and middle-income countries found that they are feasible and improve both HIV and other sexually transmitted infection prevention and care [99]. Notably, a recent systematic review and meta-analysis of digital interventions for common mental disorders in low- and middle-income countries did not include a single study conducted in Africa [108]. The review authors called for future studies “to proactively tackle these challenges and prioritize evaluating digital interventions in low-income countries, especially in Africa, considering its rapidly growing population and potential” [108]. This RCT for evaluating a digital mental health intervention, Kirabo, in Uganda and other recent [109] and ongoing protocols [110] across the continent begin to address this disparity in research.

### Limitations

There are some limitations to this protocol. The scope of activity and budget preclude a larger sample size to conduct a full analysis of intervention effectiveness and assess mediators and moderators. Additional studies will be needed to examine whether Kirabo could be implemented on a regional or national level. Further, the technological limitations in the region [23] prevent us from proposing a more interactive digital intervention. Another limitation is that the current protocol will be conducted over a relatively short period (5 weeks of intervention). Additional work could examine whether Kirabo was available for a year or longer and how the linkage to care might fluctuate as adolescents and young adults pass through developmental transitions, which could involve changes in their sexual relationships or potential levels of stress and need for counseling.

Despite these limitations, this protocol will provide preliminary estimates of Kirabo’s efficacy as a tool for promoting linkage to current HIV prevention and behavioral health care for adolescents and young adults who are susceptible to HIV. These preliminary data will serve as the basis for a larger R01 hybrid effectiveness trial [111,112], with the potential to use more advanced technology if it becomes available. This larger-scale trial would include a sufficient sample size to conduct a rigorous analysis of mediators and moderators and a longer-term follow-up to assess the impact of the mobile phone–based intervention on preventing HIV infection by promoting HIV prevention strategies and increasing linkage to care for sexual health, mental health, and alcohol use problems. Digital interventions such as Kirabo may increase access to effective biomedical HIV prevention (PrEP) and begin to address the disparities in access to behavioral health care for adolescents and young adults in Africa. Increasing access to prevention strategies and reducing factors that make adolescents and young adults susceptible to HIV acquisition can contribute to global efforts to end the HIV epidemic.

## Appendix

Multimedia Appendix 1 National Institute of Health study section Summary Statement.

## Abbreviations

ADAPT-ITT: assessment, decision, administration, production, topical experts, integration, training, testing
AUDIT-C: Alcohol Use Disorders Identification Test–Consumption
CDC: Centers for Disease Control and Prevention
CFAR: Centers for AIDS Research
CFIR: Consolidated Framework for Implementation Research
CITI: Collaborative Institutional Training Initiative
C-SSRS: Columbia Suicide Severity Rating Scale
CU: Columbia University
ESA: East and Southern Africa
GAD-7: 7-item Generalized Anxiety Disorder Questionnaire
GCP: Good Clinical Practice
ICHAD: International Center for Child Health and Development
IMB: information, motivation, and behavior
IRB: institutional review board
IVR: interactive voice response
KII: key informant interview
m4RH: Mobile 4 Reproductive Health
NIH: National Institutes of Health
NYSPI: New York State Psychiatric Institute
PHQ-4: 4-item Patient Health Questionnaire
PHQ-9: 9-item Patient Health Questionnaire
PI: principal investigator
PrEP: pre-exposure prophylaxis
PSMB: Performance and Safety Monitoring Board
RCT: randomized controlled trial
SRH: sexual and reproductive health
UNCST: Uganda National Council for Science and Technology
USSD: unstructured supplementary service data
UVRI: Uganda Virus Research Institute

## Data Availability

The quantitative data sets generated during this study will be made available in the National Institute of Mental Health (NIMH) Data Archive repository [113].
