# Correction: Mobile Phone Technology for Preventing HIV and Related Youth Health Problems, Sexual Health, Mental Health, and Substance Use Problems in Southwest Uganda (Youth Health SMS)- Protocol for a Pilot Randomized Controlled Trial

**DOI:** 10.2196/55725

**Published:** 2024-01-08

**Authors:** Philip Kreniske, Olive Imelda Namuyaba, Robert Kasumba, Phionah Namatovu, Fred Ssewamala, Gina Wingood, Ying Wei, Michele L Ybarra, Charlotte Oloya, Costella Tindyebwa, Christina Ntulo, Vincent Mujune, Larry W Chang, Claude A Mellins, John S Santelli

**Affiliations:** 1 Community Health and Social Sciences Department and The Institute for Implementation Science in Population Health, Graduate School of Public Health and Health Policy, City University of New York New York, NY United States; 2 International Center for Child Development Masaka Uganda; 3 Washington University in St Louis St Louis, MO United States; 4 Department of Sociomedical Sciences, Mailman School of Public Health, Columbia University New York, NY United States; 5 Department of Biostatistics, Mailman School of Public Health, Columbia University New York, NY United States; 6 Center for Innovative Public Health Research San Clemente, CA United States; 7 StrongMinds Uganda Kampala Uganda; 8 Malachite Center for Mental Health Kampala Uganda; 9 Department of Epidemiology, School of Medicine, Johns Hopkins Bloomberg School of Public Health Baltimore, MD United States; 10 HIV Center for Clinical and Behavioral Studies, New York State Psychiatric Institute and Columbia University New York, NY United States; 11 Heilbrunn Department of Population and Family Health, Mailman School of Public Health, Columbia University New York, NY United States

In “Mobile Phone Technology for Preventing HIV and Related Youth Health Problems, Sexual Health, Mental Health, and Substance Use Problems in Southwest Uganda (Youth Health SMS): Protocol for a Pilot Randomized Controlled Trial” (JMIR Res Protoc 2023;12:e49352), the authors noted two errors:

The affiliation of author Claude A Mellins was:

Community Health and Social Sciences Department, Graduate School of Public Health and Health Policy, City University of New York, New York, NY, United States

It has been revised to:

HIV Center for Clinical and Behavioral Studies, New York State Psychiatric Institute and Columbia University, New York, NY, United States

The affiliation of authors Charlotte Oloya, Costella Tindyebwa and Vincent Mujune was:

Malachite Center for Mental Health, Kampala, Uganda

It has been revised to:

StrongMinds Uganda, Kampala, Uganda

The correction will appear in the online version of the paper on the JMIR Publications website on January 8, 2024, together with the publication of this correction notice. Because this was made after submission to PubMed, PubMed Central, and other full-text repositories, the corrected article has also been resubmitted to those repositories.

