# Supplementary material for: Mobile Phone Technology for Preventing HIV and Related Youth Health Problems, Sexual Health, Mental Health, and Substance Use Problems in Southwest Uganda (Youth Health SMS): Protocol for a Pilot Randomized Controlled Trial
Source: JMIR Res Protoc. 2023 Dec 19;12:e49352. doi: 10.2196/49352 (PMC10762611; doi:10.2196/49352)
Supplement: Multimedia Appendix 1 [file resprot_v12i1e49352_app1.pdf]

SUMMARY STATEMENT
(Privileged Communication)

PROGRAM CONTACT:
Susannah Allison
240-627-3861
allisonsu@mail.nih.gov

Release Date: 03/30/2020
Revised Date:

Application Number: 1 K01 MH122319-01A1

Principal Investigator

KRENISKE, PHILIP

Applicant Organization: NEW YORK STATE PSYCHIATRIC INSTITUTE

Review Group: PPAH
Population and Public Health Approaches to HIV/AIDS Study Section

Meeting Date: 03/12/2020
Council: MAY 2020
Requested Start: 07/01/2020
RFA/PA: PA19-127
PCC: 9A-ASPA
Dual PCC: CV/BSV
Dual IC(s): DA, AA

Project Title: Using mobile technology to prevent HIV and related Youth Health problems: Sexual health, Mental health, and Substance use in southwest Uganda (Youth Health SMS)
SRG Action: Impact Score:22
Next Steps: Visit https://grants.nih.gov/grants/next_steps.htm
Human Subjects: 30-Human subjects involved - Certified, no SRG concerns
Animal Subjects: 10-No live vertebrate animals involved for competing appl.
Gender: 1A-Both genders, scientifically acceptable
Minority: 5A-Only foreign subjects, scientifically acceptable
Age: 6A-Children and Adults, scientifically acceptable

Project Year	Direct Costs Requested	Estimated Total Cost
1	167,126	180,496
2	169,151	182,683
3	168,279	181,741
4	165,907	179,180
5	169,186	182,721
TOTAL	839,649	906,821

ADMINISTRATIVE BUDGET NOTE: The budget shown is the requested budget and has not been adjusted to reflect any recommendations made by reviewers. If an award is planned, the costs will be calculated by Institute grants management staff based on the recommendations outlined below in the COMMITTEE BUDGET RECOMMENDATIONS section.

1K01MH122319-01A1 Kreniske, Philip

RESUME AND SUMMARY OF DISCUSSION: This candidate seeks to develop as an independent investigator with expertise in digital technology to improve adolescent and young adults' (AYA) uptake of HIV prevention, testing, and linkage to behavioral health care. In that regard, through formal coursework, directed readings, one-on-one meetings, and hands-on research activities, he plans to acquire skills in 1) longitudinal data analysis, 2) adapting mobile phone-based interventions to address multiple HIV risk factors for AYA in under-resourced settings; 3) evaluating mobile phone-based interventions for AYA in under-resourced settings; and 4) professional skills (e.g., grant and manuscript writing). For his research project, he will investigate how technology is related to known HIV risk factors and will adapt and evaluate a mobile phone-based intervention to promote HIV prevention uptake as well as mental health and substance use screening for at risk AYA in East and Southern Africa (ESA). The candidate's training plan and research are well integrated with one another and the premise for the proposed research is well justified. AYA in Eastern and Southern Africa have the highest rates of HIV infection worldwide. Moreover, mental health and substance use affect their decision making and contribute to negative outcomes about their sexual and reproductive health. This intervention, if successful, could have a very substantial impact on the health of these AYA. This is an outstanding candidate whose accomplishments to date have been impressive; the letters of support from his stellar mentors testify to their confidence in his abilities and their commitment to guiding him to his career goal. This resubmission was very responsive to prior critiques; this already strong application has been strengthened even more. The candidate has expanded his training to include more needed coursework; he has reduced the scope of his research plan to make it more manageable, and has increased the robustness of his measures of feasibility and acceptability. The application has been greatly improved. There were some remaining weaknesses; however, they were considered minor and only slightly reduced from the high enthusiasm of the committee for this application. As such, it was assessed as potentially yielding very high impact.

DESCRIPTION (provided by applicant): The proposed K01 Award will provide the candidate, Dr. Kreniske, with the skills to achieve his long-term goal of being an independent HIV investigator and an international leader who leverages digital technology to improve AYA) uptake of HIV prevention (including PrEP and HIV testing) and linkage to adolescent and young adult (behavioral health care. The candidate is a developmental psychologist with experience in qualitative and cross- sectional research, domestically and internationally. The proposed training and research experience will expand the candidate's ability to design and evaluate interventions for AYA at high risk for HIV infection and address the high priority topic of the National Institute of Health (NIH) Office of AIDS Research (OAR) of reducing the incidence of HIV. To achieve his overall goals, Dr. Kreniske will receive additional training in: (1) longitudinal data analysis for the purposes of interpreting associations between mobile phone use and protective and risk factors for HIV infection in under-resourced settings; (2) adapting mobile phone-based interventions to address multiple HIV risk factors as they relate to HIV prevention and the HIV care cascade; (3) evaluating mobile phone- based interventions for AYA in under-resourced settings using randomized controlled trials (RCT); and (4) professional skills for a successful independent research career, including grant and manuscript writing. The candidate has assembled a mentorship team of national and international leaders who will support his successful transition to independence through coursework, directed readings, one-on-one meetings, hands-on research activities, and completion of an independent research project. Dr. Kreniske's work is based in East and Southern Africa (ESA) where AYA have the highest rates of HIV infection worldwide. Mental health and substance use affect decision making about sexual and reproductive health and thus function as critical barriers to prevention of HIV infection. AYA, particularly in this socio-cultural context, face unique challenges in accessing counselors who can provide mental health and substance use assessment and treatment. To address these challenges the candidate will identify how technology is related to known HIV risk factors (e.g. sexual behaviors, mental health symptoms, substance use) and adapt and

evaluate a mobile phone-based intervention. This will serve as proof of concept for an R01 proposal to evaluate a scalable mobile phone-based intervention to reduce HIV infection by targeting at risk AYA. Research and training will occur at NYSPI/ Columbia University as well as international sites, in affiliation with two NIH-funded studies, (1) SSTAR, a study of AYA social transitions and determinants (R01HD091003; PI Santelli), which will provide secondary data from the Rakai Community Cohort Study (RCCS), and (2) SUUBI4Her (R01MH113486; PI Ssewamala), an HIV prevention study for AYA girls in Uganda that will provide secondary data on mental health. Research findings and skills obtained during the K01 will position Dr. Kreniske to become an independent investigator who can contribute to the efforts to end the HIV epidemic through innovative interventions tailored to at risk AYA.

PUBLIC HEALTH RELEVANCE: Mobile phones have changed the way adolescents and young adults (AYA) communicate, socialize, and engage in sexual relationships, with major implications for the constellation of social and biological factors that contribute to high rates of HIV infection in the transition to adulthood. AYA in East and Southern Africa (ESA) have the highest rates of HIV infection in the world, and they face unique challenges in accessing basic health information and services, including sexual health information, and counselors who can address mental health and substance abuse problems. The proposed research will generate a “proof of concept” to develop a scalable mobile phone- based intervention designed ultimately to decrease HIV incidence using mental health and substance use screening for at risk AYA in ESA.

CRITIQUE 1

Candidate: 1
Career Development Plan/Career Goals /Plan to Provide Mentoring: 1
Research Plan: 3
Mentor(s), Co-Mentor(s), Consultant(s), Collaborator(s): 1
Environment Commitment to the Candidate: 1

Overall Impact: The candidate proposes training and research activities designed to prepare him for a career as an independent research scientist who leverages digital technology to improve adolescent and young adult uptake of HIV prevention and linkage to behavioral health care. The likelihood is high that receiving the proposed training in longitudinal data analysis, adapting and evaluating mobile phone-based interventions to address multiple risk factors, and professional research skills will lead to the candidate developing an independent research career that makes important contributions to the field. The candidate’s exemplary doctoral and postdoctoral work and production of high-quality research products demonstrate his exceptional qualifications for this award, and the mentoring team and environment are well-suited to supporting his development. The candidate’s career development plan is likely to address existing gaps in his preparation, and includes a range of activities, monitoring, and support that give confidence in its likely success. His research plan is likely to maximize the potential utility of the intervention and is significant for its potential to lay the groundwork for important future research in the area of preventive interventions for adolescents and young adults. However, the likely impact of this research is reduced because relying entirely on linking youth to counselors for its impact on mental health and substance use may limit its effect, and the proposed criterion for judging efficacy is of questionable utility.

1. Candidate:

Strengths

- The candidate’s academic and research trajectory to date, including production of several first-authored publications, strongly indicates his potential to develop as an independent researcher.

- The candidate has done exemplary doctoral and postdoctoral work, as noted in the very positive letters of support.
- Through his ethnographic and other work, the candidate has demonstrated his ability to conduct high quality research.

Weaknesses

- None Noted by Reviewer

2. Career Development Plan/Career Goals & Objectives:

Strengths

- The candidate's plan is very likely to contribute to his scientific development, specifically in the areas of longitudinal data analysis, mobile intervention development, intervention evaluation, and general professional skills.
- The candidate's detailed and comprehensive description of training goals and activities corresponds well to the research plan and will address critical gaps in his otherwise excellent preparation for becoming an independent researcher.
- The plan for monitoring and evaluating progress includes detailed benchmarks and frequent opportunities for guidance and feedback from the mentoring team.

Weaknesses

- None Noted by Reviewer

3. Research Plan:

Strengths

- If successful, the planned research will address an urgent need for effective, scalable interventions to address multiple HIV risk factors among a most vulnerable population, adolescents and young adults in East and South Africa.
- Adding PrEP information and a component to address mental health and substance use issues to an existing intervention has the potential to strengthen its impact by simultaneously promoting an effective prevention method and ameliorating factors that interfere with use of preventive measures.
- Using SMS to deliver an intervention enhances feasibility.
- Described plans for adapting and pre-testing the intervention increase the likelihood that it will have the desired impact.
- Preliminary data support the premise that the targeted group is receptive to receiving health information via mobile phone.
- The proposed pilot RCT would provide essential preliminary data to serve as the basis for an R01 application.

Weaknesses

- It is unclear how Aim 1 activities designed to assess the impact of mobile phone ownership on HIV incidence is relevant to the work planned in Aims 2 and 3, as the intervention to be developed is not intended to reduce phone use.

- It appears that the planned intervention's impact on mental health and substance use may be entirely dependent on users linking to school counselors and then receiving effective intervention from those counselors. However, the application does not make a strong case for expecting that linkage will have the desired impact on mental health and substance use. If the SMS intervention itself is expected to have a direct effect on these issues, the mechanism and premise for such an effect is not clear.
- The candidate's statement that technological limitations prevent proposing an interactive mental health and substance use intervention seems to underestimate the potential for SMS as a delivery platform for intervention. A review of literature in this area would have been useful to clarify the point.
- The primary endpoint (as described in 4.C.6.1) will be "the binary indicator whether there is an increase in any of these HIV prevention strategies following the intervention; PrEP uptake, number of HIV tests within study period, and safer sexual behaviors." This definition is vague, and outcomes judged by this criterion are not likely to be informative.
- The power analysis based on the primary endpoint offers no justification for the expected proportion who would "increase their HIV prevention strategies" either with or without intervention.

4. Mentor(s), Co-Mentor(s), Consultant(s), Collaborator(s):

Strengths

- The mentoring team is highly qualified to provide support in the career development and research activities described in this application.
- The primary mentor (Santelli) currently serves as the candidate's mentor in the postdoctoral fellowship.

Weaknesses

- None Noted by Reviewer

5. Environment and Institutional Commitment to the Candidate:

Strengths

- Letters from the department chair, mentors, and others demonstrate a strong commitment to the candidate's development as an independent researcher.
- Resources adequate to support the candidate's development and conduct of the research project are currently in place.

Weaknesses

- None Noted by Reviewer

Study Timeline:

Strengths

- The planned timeline describes a logical progression of activities that should be feasible to carry out within the time frame of the project.

Weaknesses

- None Noted by Reviewer

Protections for Human Subjects:

Acceptable Risks and Adequate Protections

- Potential sources of risk are appropriately minimized, and overall risk to participants is low.

Data and Safety Monitoring Plan (Applicable for Clinical Trials Only):

Acceptable

Inclusion Plans:

- Sex/Gender: Distribution justified scientifically
- Race/Ethnicity: Distribution justified scientifically
- For NIH-Defined Phase III trials, Plans for valid design and analysis: Not applicable
- Inclusion/Exclusion Based on Age: Distribution justified scientifically
- Plans for specific targeting of youth age 15-19 are appropriate due to vulnerability.

Vertebrate Animals:

Not Applicable (No Vertebrate Animals)

Biohazards:

Not Applicable (No Biohazards)

Resubmission:

- The research plan has been scaled back with regard to number of subjects for Aim 3 key informant interviews, and the current plan seems feasible. Formal training in mHealth has been added. Details regarding the intervention to be adapted have been added.

Training in the Responsible Conduct of Research:

Acceptable

Comments on Format (Required):

- Combination of directed study, coursework, and training workshops.

Comments on Subject Matter (Required):

- Training will be broad, covering all required topics.

Comments on Faculty Participation (Required; not applicable for mid- and senior-career awards):

- Mentors will engage regularly in discussions of ethics.

Comments on Duration (Required):

- Exceeds criterion.

Comments on Frequency (Required):

- Exceed criterion.

Resource Sharing Plans:

Not Applicable (No Relevant Resources)

Authentication of Key Biological and/or Chemical Resources:

Not Applicable (No Relevant Resources)

Budget and Period of Support:

Recommend as Requested:

CRITIQUE 2

Candidate: 3
Career Development Plan/Career Goals /Plan to Provide Mentoring: 2
Research Plan: 4
Mentor(s), Co-Mentor(s), Consultant(s), Collaborator(s): 1
Environment Commitment to the Candidate: 1

Overall Impact*:* This is a well-written application by a strong candidate. Although relatively new to the HIV prevention and treatment field, he has an excellent publication record and is highly committed to this science. The mentorship team and research and field environments are exceptional. The career development plan has been significantly strengthened in the resubmission, and it is closely aligned with the research plan. The research plan, which addresses a highly significant population with an innovative yet scientifically grounded intervention, has also been clarified and strengthened in the resubmission. The prospects for successful completion of the pilot study and submission of an R01 are raised considerably. There are some moderate weaknesses in the research plan, but overall impact is likely to be high.

1. Candidate:

Strengths

- The candidate was awarded his PhD. In 2016. His publication record since then is impressive.
- Letters of recommendation are highly supportive.
- Although relatively new to the HIV field, Dr. Kreniske has conducted advanced work for the research plan in Uganda.

Weaknesses

- The development and research plans are ambitious given the candidate's limited prior experience in this field.

2. Career Development Plan/Career Goals & Objectives:

Strengths

- The development plan focuses on longitudinal data analysis, adaptation and evaluation of behavioral interventions, and career skills for publishing and grantsmanship.
- The development plan is aligned with the research plan.

- The resubmission includes additional rigorous training in statistics, mHealth, and RCT design and execution.
- The development plan includes a challenging publication timeline.

Weaknesses

- None Noted by Reviewer

3. Research Plan:

Strengths

- The recruitment and retention plan is supported by existing infrastructure in Uganda; parents will receive invitations for their children to participate through schools.
- The m4RH intervention to be adapted was shown to be efficacious in Kenya and Tanzania.
- The adapted intervention will Incorporate PrEP information and mental health and alcohol use measures and components.
- The pilot trial includes 140 youth randomized to the intervention or control conditions; this should provide sufficient data for feasibility and acceptability assessments as well as preliminary efficacy evaluation.
- The resubmission now includes completion and evaluation of the pilot trial by the middle of Year 4, such that an R01 can be considered and submitted by the end of Year 4. The intervention will use the same phone technology platform as the m4RH now being rolled out (but not evaluated) in Uganda, presumably for an older age group.
- The HBM theoretical model, with the addition of peer influence, appears quite appropriate for the target population.
- The outcome measures and analyses are well designed.

Weaknesses

- Data on mental health problems among AYA are not provided.
- The measures of mental distress appear to be those that have been well-validated in high income Western countries. In this context, including in the ongoing research by the mentors working in Uganda, the measures all appear to be exploratory. No prior or proposed validation procedures are apparent in the application.

4. Mentor(s), Co-Mentor(s), Consultant(s), Collaborator(s):

Strengths

- The mentor team includes the candidate's current mentor as primary mentor, together with an extraordinarily qualified team of experts in both the development plan and the Uganda-based research plan.
- The letters of support are very strong.
- The mentors have a history of collaboration as well as mentorship.

Weaknesses

- None Noted by Reviewer

5. Environment and Institutional Commitment to the Candidate:

Strengths

- The research environment at Columbia is excellent.
- The field research infrastructure in Uganda is in place.
- There is institutional commitment to the candidate, including the appointment to a faculty position without regard to the success of the K award.

Weaknesses

- None Noted by Reviewer

Study Timeline:

Strengths

- Appropriate

Weaknesses

- None Noted by Reviewer

Protections for Human Subjects:

Acceptable Risks and Adequate Protections

Data and Safety Monitoring Plan (Applicable for Clinical Trials Only):

Acceptable

Inclusion Plans:

- Sex/Gender: Distribution justified scientifically
- Race/Ethnicity: Distribution justified scientifically
- For NIH-Defined Phase III trials, Plans for valid design and analysis: Not applicable
- Inclusion/Exclusion Based on Age: Distribution justified scientifically
- Foreign men and women ages 15-19

Vertebrate Animals:

Not Applicable (No Vertebrate Animals)

Biohazards:

Not Applicable (No Biohazards)

Resubmission:

- Responsive, including changes recommended to the training and research plans

Training in the Responsible Conduct of Research:

Acceptable

Comments on Format (Required):

- Directed readings, coursework and mentor meetings

Comments on Subject Matter (Required):

- Digital technology, youth sexual health and mental health

Comments on Faculty Participation (Required; not applicable for mid- and senior-career awards):

- Regular meetings with mentors

Comments on Duration (Required):

- Years 1-4

Comments on Frequency (Required):

- Mentor meetings at least every 4 months, course work and workshops approximately once per year

Resource Sharing Plans:

Not Applicable (No Relevant Resources)

Authentication of Key Biological and/or Chemical Resources:

Not Applicable (No Relevant Resources)

Budget and Period of Support:

Recommend as Requested:

CRITIQUE 3

Candidate: 2
Career Development Plan/Career Goals /Plan to Provide Mentoring: 2
Research Plan: 3
Mentor(s), Co-Mentor(s), Consultant(s), Collaborator(s): 1
Environment Commitment to the Candidate: 1

Overall Impact: This K01 resubmission is very responsive to reviewer comments. Dr. Kreniske is an exceptional candidate in HIV prevention research among adolescent and young adults (AYA) in southwest Uganda. Dr. Kreniske is completing his postdoc at the HIV Center at New York State Psychiatric Institute/Columbia University. There is strong institutional and mentor support for the candidate, who has been productive in his research and publications thus far. The candidate's long-term career goal is to become an independent investigator in HIV prevention with AYAs in under-resources settings. The candidate's research plan maps onto the training plan, which is comprehensive. The proposed research will randomize 140 AYA to an mHealth intervention versus a control. The primary outcome is increased HIV prevention strategies post-intervention. It is unclear what the control group is, however. If successful, the research may yield a potentially high public health impact, in that this intervention could be scaled-up and adapted for other countries.

1. Candidate:

Strengths

- Candidate is a postdoc at the HIV Center for Clinical and Behavioral Studies at NYSPI and Columbia University and lectures in PopFam at Columbia.
- Reference letters from the candidate's mentors and collaborators are highly supportive.
- The candidate currently has 7 first-authored manuscripts with several papers under review.
- The candidate was a silver medalist for the NYAS James McKeen Cattell Dissertation Award.
- The candidate's work in East and Southern Africa (ESA) has examined the impact of disparities on health outcomes and this work has set the stage for the training grant.
- The candidate has conducted research on mHealth interventions and has more recently focused his area in HIV prevention and linkage to care, which led to the development of this application. The K01 would help to establish the candidate in the HIV prevention field, specifically in mHealth interventions with AYA.

Weaknesses

- No concerns.

2. Career Development Plan/Career Goals & Objectives:

Strengths

- Career development goals are clearly defined and align with the research plan and future R01 submission.
- The proposed trips to Uganda will enhance the learning experience; it is also important for a PI to be present at study locations.
- The candidate has a well-balanced training plan that covers broad topics such technology and statistics and across various formats, including formal learning, field experience, and mentor-related training.
- The distribution of effort, and types of training, across activities is excellent.

Weaknesses

- No major concerns.

3. Research Plan:

Strengths

- The proposed study is highly significant, as East and Southern Africa have the highest rate of incident HIV infections globally, with AYA at greatest risk. Text messaging is widely used and is a viable medium for interventions, including sexual health education via text messaging.
- The candidate proposes to adapt and evaluate an evidence-based mobile phone-delivered sexual health program, to include PrEP information, as well as mental health and alcohol use screening and linkage to school-based counselors for AYA in southwest Uganda.
- This work leverages established structure developed by mentor Dr. Ssewamala.
- FHI 360 adapted m4RH for 10-24 AYA in Uganda to address their sexual and reproductive health information needs; adapting m4RH for HIV prevention information is a logical next step.
- The research Aims are clear and the research plan is nicely described for each step of ADAPT-ITT as well as study implementation.

- Table 5 succinctly describes the proposed ADAPT-ITT methodology by study year, phase, and methodology.
- The primary outcome is an increase in any of the HIV prevention strategies post-intervention, including PrEP uptake, number of HIV tests within study period, and safer sexual behaviors (cites in Table 7).
- The mixed methods approach will yield rich data.
- The candidate added new feasibility measures plus a threshold for intervention feasibility and acceptability.

Weaknesses

- There is no information about the control group.
- Uptake of HIV prevention strategies is more clearly defined; however, there is limited information regarding how HIV prevention information will actually be incorporated into the intervention.
- Theatre testing now includes 12 KII (up from 6) which should be enough to assess preliminary feasibility and acceptability.
- It would be helpful to list the outcome variables in Table 7, rather than list citations.

4. Mentor(s), Co-Mentor(s), Consultant(s), Collaborator(s):

Strengths

- The candidate has assembled a stellar multidisciplinary mentorship team that has a history of collaboration.
- Primary mentor, Dr. Santelli, is an expert in AYA health and medicine, social determinants and social transitions, sexual health, and HIV risk factors.
- Co-mentor, Dr. Mellins, is an expert in AYA development and mental health, HIV prevention interventions, and global mental health.
- Co-mentor, Dr. Wingood, has expertise in implementation science and intervention adaptation for health and HIV-related interventions; and has developed the ADAPT_ITT model to facilitate adapting evidence-based HIV interventions to diverse populations.
- The advisers have expertise in fields relevant to the candidate's career development plan, including advanced longitudinal data analysis and RCT evaluation (Wei), design and evaluation of HIV prevention RCTs in under-resourced settings (Ssewamala), design of mHealth interventions in under-resourced settings (Chang) and mHealth for AYA (Ybarra).

Weaknesses

- None Noted by Reviewer

5. Environment and Institutional Commitment to the Candidate:

Strengths

- The HIV Center at NYSPI and Columbia University have outstanding resources, and it is clear from the letters of support that the candidate will be well-supported by his mentorship team.

Weaknesses

- No concerns.

Study Timeline:

Strengths

- Well-detailed and realistic; more time has been set aside for evaluation, grant submission and manuscript preparation.

Weaknesses

- No concerns.

Protections for Human Subjects:

Acceptable Risks and Adequate Protections

Data and Safety Monitoring Plan (Applicable for Clinical Trials Only):

Acceptable

Inclusion Plans:

- Sex/Gender: Distribution justified scientifically
- Race/Ethnicity: Distribution justified scientifically
- For NIH-Defined Phase III trials, Plans for valid design and analysis: Scientifically acceptable
- Inclusion/Exclusion Based on Age: Distribution justified scientifically

Vertebrate Animals:

Not Applicable (No Vertebrate Animals)

Biohazards:

Not Applicable (No Biohazards)

Training in the Responsible Conduct of Research:

Acceptable

Comments on Format (Required):

- RCR training format via one-on-one meetings with mentors and guided reading, as well as videoconferencing and a formal ethics training.

Comments on Subject Matter (Required):

- Content will focus on behavioral research, the development mHealth technology,
- and research with youth in under-resourced international settings.

Comments on Faculty Participation (Required; not applicable for mid- and senior-career awards):

- Dr. Santelli will provide guidance on ethical issues related to adolescent sexual health (Years 1-4). Dr. Mellins provide guidance on ethical training focused on evaluation and treatment of mental health among global youth. Dr. Ybarra will lead training on digital technology and ethical issues.

Comments on Duration (Required):

- Training is covered through years 1-4.

Comments on Frequency (Required):

- Monthly meetings with mentor and co-mentor.

Resource Sharing Plans:
Acceptable

Authentication of Key Biological and/or Chemical Resources:
Not Applicable (No Relevant Resources)

Budget and Period of Support:
Recommend as Requested:

THE FOLLOWING SECTIONS WERE PREPARED BY THE SCIENTIFIC REVIEW OFFICER TO SUMMARIZE THE OUTCOME OF DISCUSSIONS OF THE REVIEW COMMITTEE, OR REVIEWERS' WRITTEN CRITIQUES, ON THE FOLLOWING ISSUES:

PROTECTION OF HUMAN SUBJECTS: ACCEPTABLE

INCLUSION OF WOMEN PLAN: ACCEPTABLE

INCLUSION OF MINORITIES PLAN: ACCEPTABLE

INCLUSION ACROSS THE LIFESPAN: ACCEPTABLE

COMMITTEE BUDGET RECOMMENDATIONS: The budget was recommended as requested.

Footnotes for 1 K01 MH122319-01A1; PI Name: Kreniske, Philip

NIH has modified its policy regarding the receipt of resubmissions (amended applications).See Guide Notice NOT-OD-18-197 at https://grants.nih.gov/grants/guide/notice-files/NOT-OD-18-197.html. The impact/priority score is calculated after discussion of an application by averaging the overall scores (1-9) given by all voting reviewers on the committee and multiplying by 10. The criterion scores are submitted prior to the meeting by the individual reviewers assigned to an application, and are not discussed specifically at the review meeting or calculated into the overall impact score. Some applications also receive a percentile ranking. For details on the review process, see http://grants.nih.gov/grants/peer_review_process.htm#scoring.

MEETING ROSTER

Population and Public Health Approaches to HIV/AIDS Study Section
AIDS and Related Research Integrated Review Group
CENTER FOR SCIENTIFIC REVIEW
PPAH
03/12/2020 - 03/13/2020

Notice of NIH Policy to All Applicants: Meeting rosters are provided for information purposes only. Applicant investigators and institutional officials must not communicate directly with study section members about an application before or after the review. Failure to observe this policy will create a serious breach of integrity in the peer review process, and may lead to actions outlined in NOT-OD-14-073 at https://grants.nih.gov/grants/guide/notice-files/NOT-OD-14-073.html and NOT-OD-15-106 at https://grants.nih.gov/grants/guide/notice-files/NOT-OD-15-106.html, including removal of the application from immediate review.

CHAIRPERSON(S)

ALLEN, SUSAN A, MD
PROFESSOR
DEPARTMENT OF PATHOLOGY
AND LABORATORY MEDICINE
SCHOOL OF MEDICINE
EMORY UNIVERSITY
ATLANTA, GA 30322

MEMBERS

ABUOGI, LISA LYNN, MD *
ASSOCIATE PROFESSOR
DEPARTMENT OF PEDIATRICS
SCHOOL OF MEDICINE
UNIVERSITY OF COLORADO, DENVER
AURORA 80045

BALASUBRAMANIAN, RAJI, AB, DSC *
ASSOCIATE PROFESSOR
DEPARTMENT OF BIOSTATISTICS AND EPIDEMIOLOGY
SCHOOL OF PUBLIC HEALTH AND HEALTH SCIENCES
UNIVERSITY OF MASSACHUSETTS
AMHERST, MA 01003

BASS, SARAH B, MPH, PHD *
ASSOCIATE PROFESSOR
DEPARTMENT OF SOCIAL AND BEHAVIORAL SCIENCES
COLLEGE OF PUBLIC HEALTH
TEMPLE UNIVERSITY
PHILADELPHIA, PA 19122

BAUERMEISTER, JOSE ARTURO, PHD
PROFESSOR
DEPARTMENT OF FAMILY AND COMMUNITY HEALTH
SCHOOL OF NURSING
UNIVERSITY OF PENNSYLVANIA
PHILADELPHIA, PA 19104

BAUMAN, LAURIE J, PHD
PROFESSOR
DEPARTMENT OF PEDIATRICS
ALBERT EINSTEIN COLLEGE OF MEDICINE
BRONX, NY 10461

BLUTHENTHAL, RICKY N, PHD *
PROFESSOR
DEPARTMENT OF PREVENTIVE MEDICINE
KECK SCHOOL OF MEDICINE
UNIVERSITY OF SOUTHERN CALIFORNIA
LOS ANGELES, CA 90033

BOEKELOO, BRADLEY O, PHD *
PROFESSOR
DEPARTMENT OF BEHAVIORAL AND COMMUNITY HEALTH
SCHOOL OF PUBLIC HEALTH
UNIVERSITY OF MARYLAND
COLLEGE PARK, MD 20742

BOODRAM, BASMATTEE, PHD *
ASSOCIATE PROFESSOR
DIVISION OF COMMUNITY HEALTH SCIENCES
SCHOOL OF PUBLIC HEALTH
UNIVERSITY OF ILLINOIS AT CHICAGO
CHICAGO, IL 60612

BROWN, MONIQUE JANIEL, PHD *
ASSISTANT PROFESSOR
DEPARTMENT OF EPIDEMIOLOGY AND BIOSTATISTICS
ARNOLD SCHOOL OF PUBLIC HEALTH
UNIVERSITY OF SOUTH CAROLINA
COLUMBIA, SC 29208

CARNEGIE, NICOLE BOHME, PHD *
ASSISTANT PROFESSOR
DEPARTMENT OF MATHEMATICAL SCIENCES
MONTANA STATE UNIVERSITY
BOZEMAN, MT 59715

CHARLEBOIS, EDWIN DUNCAN, PHD *
PROFESSOR
DEPARTMENT OF MEDICINE
SCHOOL OF MEDICINE
UNIVERSITY OF CALFORNIA, SAN FRANCISCO
SAN FRANCISCO, CA 94105

CHEN, YING QING, PHD *
FULL MEMBER
PROGRAM IN BIOSTATISTICS
VACCINE AND INFECTIOUS DISEASE DIVISION
FRED HUTCHINSON CANCER RESEARCH CENTER
SEATTLE, WA 98109

CLATTS, MICHAEL C, PHD *
PROFESSOR
DEPARTMENT OF HEALTH SERVICES ADMINISTRATION
AND EVALUATION
SCHOOL OF PUBLIC HEALTH
UNIVERSITY OF PUERTO RICO
SAN JUAN, PR 00936

DARBES, LYNAE A, PHD *
ASSOCIATE PROFESSOR
DEPARTMENT OF HEALTH BEHAVIOR
AND BIOLOGICAL SCIENCES
SCHOOL OF NURSING
UNIVERSITY OF MICHIGAN
ANN ARBOR, MI 48109

DAVIES, SUSAN L, PHD *
PROFESSOR
DEPARTMENT OF HEALTH BEHAVIOR
SCHOOL OF PUBLIC HEALTH
UNIVERSITY OF ALABAMA AT BIRMINGHAM
BIRMINGHAM, AL 35294

DERMEN, KURT H, PHD *
SENIOR RESEARCH SCIENTIST
RESEARCH INSTITUTE ON ADDICTIONS
UNIVERSITY AT BUFFALO
STATE UNIVERSITY OF NEW YORK
BUFFALO, NY 14203-1014

EATON, LISA A, PHD *
ASSOCIATE PROFESSOR
DEPARTMENT OF HUMAN DEVELOPMENT
AND FAMILY STUDIES
COLLEGE OF LIBERAL ARTS AND SCIENCES
UNIVERSITY OF CONNECTICUT
STORRS, CT 06029

FLYNN, PATRICK M, PHD, MA, BA *
PROFESSOR AND DIRECTOR
INSTITUTE OF BEHAVIORAL RESEARCH
DEPARTMENT OF PSYCHOLOGY
TEXAS CHRISTIAN UNIVERSITY
FT WORTH, TX 76109

FUJIMOTO, KAYO, PHD
ASSOCIATE PROFESSOR
DEPARTMENT OF HEALTH PROMOTIONS
AND BEHAVIORAL SCIENCES
SCHOOL OF PUBLIC HEALTH
UNIVERSITY OF TEXAS AT HOUSTON
HOUSTON, TX 77030

HABERER, JESSICA ELIZABETH, MD
ASSOCIATE PROFESSOR
DEPARTMENT OF MEDICINE
MASSACHUSETTS GENERAL HOSPITAL
BOSTON, MA 02114

HECKMAN, TIMOTHY GLENN, PHD *
PROFESSOR AND ASSOCIATE DEAN
DEPARTMENT OF HEALTH PROMOTION AND BEHAVIOR
COLLEGE OF PUBLIC HEALTH
UNIVERSITY OF GEORGIA
ATHENS, GA 30602

HIRSHFIELD, SABINA, PHD *
PRINCIPAL RESEARCH SCIENTIST
SUNY DOWNSTATE HEALTH SCIENCES UNIVERSITY
BROOKLYN, NY 11203

KERSHAW, TRACE S, PHD
PROFESSOR
CENTER FOR INTERDISCIPLINARY RESEARCH ON AIDS
DEPARTMENT OF EPIDEMIOLOGY
SCHOOL OF PUBLIC HEALTH
YALE UNIVERSITY
NEW HAVEN, CT 06510

KURTZ, STEVEN P, PHD *
PROFESSOR AND DIRECTOR
CENTER FOR APPLIED RESEARCH ON SUBSTANCE USE
AND HEALTH DISPARITIES
DEPARTMENT OF JUSTICE AND HUMAN SERVICES
NOVA SOUTHEASTERN UNIVERSITY
CORAL GABLES, FL 33134

MCCLELLAND, RAYMOND SCOTT, MD
PROFESSOR
DEPARTMENTS OF MEDICINE, EPIDEMIOLOGY,
AND GLOBAL HEALTH
UNIVERSITY OF WASHINGTON
SEATTLE, WA 98104

MCKAY, VIRGINIA R, PHD *
RESEARCH ASSISTANT PROFESSOR
CENTER FOR PUBLIC HEALTH SYSTEMS SCIENCE
WASHINGTON UNIVERSITY AT ST. LOUIS
ST. LOUIS, MO 63130

MCMAHON, JAMES M, PHD
ASSOCIATE PROFESSOR
SCHOOL OF NURSING
UNIVERSITY OF ROCHESTER MEDICAL CENTER
ROCHESTER, NY 14642

MEEK, ERIN, DRPH
SENIOR RESEARCH SCIENTIST
AIDS OFFICE
SAN FRANCISCO DEPARTMENT OF PUBLIC HEALTH
SAN FRANCISCO, CA 94102

MUESSIG, KATHRYN E, PHD *
ASSOCIATE PROFESSOR
DEPARTMENT OF HEALTH BEHAVIOR
GILLINGS SCHOOL OF GLOBAL PUBLIC HEALTH
UNIVERSITY OF NORTH CAROLINA AT CHAPEL HILL
CHAPEL HILL, NC 27599

NAAR, SYLVIE, PHD *
PROFESSOR
DEPARTMENT OF BEHAVIORAL SCIENCES
AND SOCIAL MEDICINE
COLLEGE OF MEDICINE
FLORIDA STATE UNIVERSITY
TALLAHASSEE, FL 32304

ONDERSMA, STEVEN J, PHD *
PROFESSOR
DEPARTMENT OF PSYCHIATRY
AND BEHAVIORAL NEUROSCIENCE
SCHOOL OF MEDICINE
WAYNE STATE UNIVERSITY
DETROIT, MI 48236

PADILLA, MARK B, PHD *
ASSOCIATE PROFESSOR
DEPARTMENT OF GLOBAL AND SOCIOCULTURAL STUDIES
SCHOOL OF INTERNATIONAL AND PUBLIC AFFAIRS
FLORIDA INTERNATIONAL UNIVERSITY
MIAMI, FL 33199

ROY, JASON A, PHD *
PROFESSOR AND CHAIR
DEPARTMENT OF BIOSTATISTICS
AND EPIDEMIOLOGY
SCHOOL OF PUBLIC HEALTH
RUTGERS UNIVERSITY
PISCATAWAY, NJ 08854

SALEMI, MARCO, PHD *
PROFESSOR
DEPARTMENT OF PATHOLOGY, IMMUNOLOGY,
AND LABORATORY MEDICINE
COLLEGE OF MEDICINE
UNIVERSITY OF FLORIDA
GAINESVILLE , FL 32610

SHERMAN, SUSAN GAIL, PHD
PROFESSOR
DEPARTMENT OF HEALTH, BEHAVIOR, AND SOCIETY
SCHOOL OF PUBLIC HEALTH
JOHNS HOPKINS UNIVERSITY
BALTIMORE, MD 21205

STARKS, TYREL J, PHD *
ASSOCIATE PROFESSOR
DEPARTMENT OF PSYCHOLOGY
HUNTER COLLEGE
NEW YORK, NY 10065

SWEAT, MICHAEL D, PHD
PROFESSOR
DEPARTMENT OF PSYCHIATRY
AND BEHAVIORAL SCIENCES
MEDICAL UNIVERSITY OF SOUTH CAROLINA
CHARLESTON, SC 29407

WEINHARDT, LANCE S, PHD *
PROFESSOR
DEPARTMENT OF COMMUNITY
AND BEHAVIORAL HEALTH PROMOTION
JOSEPH J ZILBER SCHOOL OF PUBLIC HEALTH
UNIVERSITY OF WISCONSIN, MILWAUKEE
MILWAUKEE, WI 53201

WITTE, SUSAN S, PHD
PROFESSOR
SCHOOL OF SOCIAL WORK
COLUMBIA UNIVERSITY
NEW YORK, NY 10027

YOUNG, APRIL MARIE, PHD *
ASSOCIATE PROFESSOR
DEPARTMENT OF EPIDEMIOLOGY
COLLEGE OF PUBLIC HEALTH
UNIVERSITY OF KENTUCKY
LEXINGTON, KY 40536

ZEA, MARIA CECILIA, PHD *
PROFESSOR
DEPARTMENT OF PSYCHOLOGY
COLLEGE OF ARTS AND SCIENCES
GEORGE WASHINGTON UNIVERSITY
WASHINGTON, DC 20052

MAIL REVIEWER(S)

ALEXANDRE, PIERRE K, PHD
DIRECTOR AND ASSOCIATE PROFESSOR
DEPARTMENT OF MANAGEMENT PROGRAMS
HEALTH ADMINISTRATION
COLLEGE OF BUSINESS
FLORIDA ATLANTIC UNIVERSITY
BOCA RATON, FL 33431

SCIENTIFIC REVIEW OFFICER

GUERRIER, JOSE H, PHD
SCIENTIFIC REVIEW OFFICER
CENTER FOR SCIENTIFIC REVIEW
NATIONAL INSTITUTES OF HEALTH
BETHESDA, MD 20892

EXTRAMURAL SUPPORT ASSISTANT

STROTHERS, DIARA
EXTRAMURAL SUPPORT ASSISTANT
CENTER FOR SCIENTIFIC REVIEW
NATIONAL INSTITUTES OF HEALTH
BETHESDA, MD 20892

* Temporary Member. For grant applications, temporary members may participate in the entire meeting or may review only selected applications as needed.

Consultants are required to absent themselves from the room during the review of any application if their presence would constitute or appear to constitute a conflict of interest.
